# Soft Targets: Nurses and the Pharmaceutical Industry

**DOI:** 10.1371/journal.pmed.0050005

**Published:** 2008-02-05

**Authors:** Annemarie Jutel, David B Menkes

## Abstract

The nursing literature has yet to pay much attention to the expansive reach of the pharmaceutical industry into the nursing profession.

“Our Special Projects Division produces a wide portfolio of therapy and topic-specific materials…available for sponsorship by companies who wish to promote their products or services, while visibly supporting nurse education,” [1] invites *Nursing Standard*, the journal of the United Kingdom Royal College of Nursing.

The commercial sponsorship of nursing education exemplified by this advertisement reflects the fact that nurses have increasing power to choose products and services, and to influence choices made by medical and other colleagues [2]. Prescription pharmaceuticals provide a notable example of how nurses have become, as proclaimed above, a desirable target for a powerful industry.

This industry has been robustly critiqued in the medical literature for exploiting patients and doctors using a range of techniques: direct-to-consumer advertising (DTCA) [3–6], sponsored teaching materials [7], advertising in professional media [8,9], research funding [10–14], ghost writing [15,16], gifts, free meals, and travel [7,13,17].

The nursing literature has yet to pay much attention to the expansive reach of the pharmaceutical industry into the nursing profession. In this article, we examine some of the key literature on the influence of drug companies upon nurses, consider the limitations of this literature, and define a strategy for heightening awareness and strengthening the skills of nurses to manage the impact of commercial interests.

## Our Literature Search

We searched MedLine (http://www.ncbi.nlm.nih.gov/pubmed/) and CINAHL (http://www.cinahl.com/prodsvcs/cinahldb.htm) databases without date restriction in May 2007, using the terms “pharmaceutical industry,” “drug sales,” “direct-to-consumer,” and “pharmaceutic*,” and restricted to nursing journals. We searched the same terms without the nursing journal restriction, combined with the truncated search terms “nurs*” and prescri*.” A search combining the term “nurs*” with “gift,” a hand search, and references from colleagues completed our research.

We included all articles making reference to nursing's relationship to drug companies and those that included the perspectives of the pharmaceutical industry on this issue. As our intention was to understand the field, we examined all types of articles, from empirical research papers to pure opinion.

We found 32 articles that met our inclusion criteria. Of these, seven were empirical studies (summarised in Table 1), two were theoretical, using anthropological or ethical frameworks to describe the implications of the gift exchange, and 23 were perspectives, commentaries, opinions, and non-systematic (narrative) reviews. There were 16 articles from nursing journals, nine from nurse practitioner journals, two from medical journals, and five from multi-disciplinary journals.

**Table 1:**
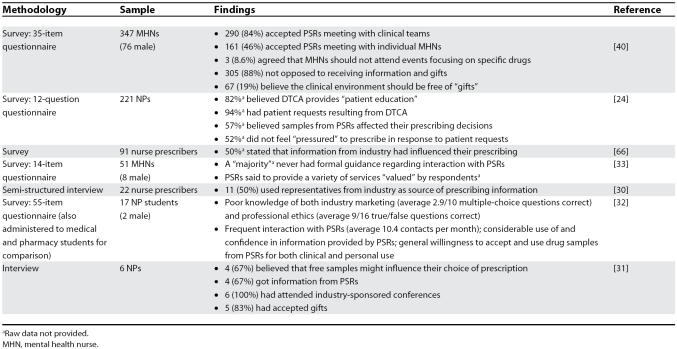
Summary of Empirical Findings Regarding Pharmaceutical Industry Influence on Nurses

From these 32 articles, we identified and grouped topics and concerns, and positioned them relative to debates in the medical literature about the influence of the pharmaceutical industry upon patient and professional education, gift giving, DTCA, provision of free drug samples, and other determinants of prescribing practice.

## Our Findings

### Balance of criticism versus support.

Given the vociferous debate in the medical literature, we anticipated positioned papers that would take clear stances for or against the involvement of the pharmaceutical industry in the nursing profession. Such a dichotomy was not evident in the articles we located. Of the 32 articles, 13 expressed or reported serious concerns about the role of the pharmaceutical industry, and four were clearly industry-friendly; these are explored in greater depth in Table 2. The remaining publications either expressed mild concern about the pharmaceutical industry, viewed the support of the industry as generally favourable, or identified both the harms and benefits of the industry's involvement in health care.

**Table 2:**
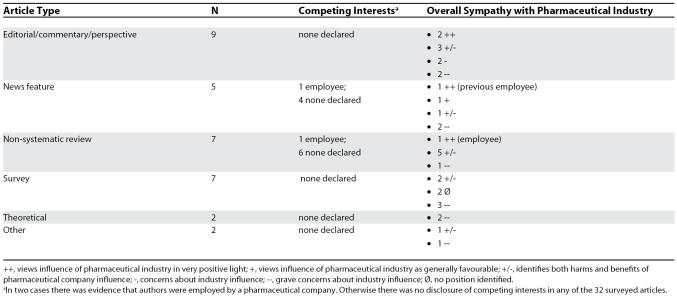
Summary of Article Positions and Competing Interests

### Direct-to-consumer advertising.

Seven articles address DTCA, three of these providing overviews and identifying consequent problems for patient–clinician communication. The overviews draw upon the medical literature, and lament the scant research available to assess the impact of DTCA on nurses [18–20], but offer limited criticism. One of these overviews focused on presenting a balanced report of the benefits and harms of DTCA, presenting a number of arguments both for and against the practice, but concluding with the view that DTCA might benefit patients by prompting them to seek medical attention, and suggesting that a “balance” is required [19].

An opinion piece, written by health advocate Charles Inlander for a nursing economics journal, praises DTCA as an antidote to medicine's self-interested reluctance to share information [21]. Another, written by employees of the industry, speaks of guarding the line between promotion and education, but not surprisingly concludes that the industry has an important role to play in patient education—one that these authors hope will expand [22]. Three articles encourage health care professionals to work *with* the pharmaceutical industry to promote accurate patient information, and not to be predisposed against DTCA [20,23,24].

### Professional education.

Many of the articles (ten out of 31) draw attention to the substantial role that information from the pharmaceutical industry plays in the education of nurses [22,25–33]. Sponsored professional education, drug samples and information, small gifts, and patient services are portrayed as beneficial, even though caution is advised in their use—unethical behaviour is cast as possible, but exceptional [28,34]. Lynore DeSilets, a nursing educator, recommends robust professional guidelines and accreditation as safeguards [27].

### Samples and gifts from, or contacts with, pharmaceutical sales representatives.

Fifteen of the articles consider pharmaceutical sales representatives (PSRs) and/or their provision of drug samples to prescribing nurses. In an advice column to nurse practitioners (NPs), lawyer Cathy Klein acknowledges evidence that provision of samples influences medical prescribing, and increases rather than decreases costs, but also views pharmaceutical representatives as an important source of practical guidance and information for nurses [35]. Bambi Alexander-Banys, in a guest editorial for the *Journal of Pediatric Health Care*, starts by acknowledging and appreciating the pharmaceutical industry's support of NPs, and then criticises the industry for failing to court NPs or make reference to them in DTCA as they do doctors. This, the author suggests, reinforces traditional perceptions of the latter as the credible provider of patient care, to the detriment of the NP role [36].

In an editorial exchange in *The Nurse Practitioner*, student family nurse practitioner Sarah Sidiqi argues that NPs may be unwitting victims of the pharmaceutical industry's commercial agenda [37], but receives a sceptical response from the editor, who questions the evidence that NPs have been approached inappropriately in the ways that doctors have, and points out that NPs are generally ignored by the industry [38]. A pilot interview study of six NPs found that all believed pharmaceutical companies influenced their prescribing, with both positive and negative consequences [31]. A survey of 221 oncology NPs found that over half (57%) of respondents viewed the provision of sample drugs as having affected their prescribing choice [24].

Many articles conclude that small gifts from PSRs are acceptable. Jill Davies and Steve Hemingway, both nursing educators, suggest such gifts should not exceed UK£5 (~US$10) [26], while an editor of *The Nurse Practitioner*, Marilyn Edmunds, as well as clinical nurse specialist Patricia O'Malley, see US$100 as the top range for such gifts [28,39]. Not surprisingly, a “medical writer” with “12 years' experience in pharmaceutical sales training” writing in *Advance for Nurse Practitioners* refers to promotional objects as a normal part of professional practice and fails to offer any critical consideration of the gift or its consequences [29]. Melodie Young, president of the Dermatology Nurses' Association, mirrors this approach in her article, which promotes PSRs as providing important support for nurses in *Dermatology Nursing* [34].

Michael Monaghan and colleagues used a cross-sectional survey to determine that NP students (*n* = 17) had both more positive attitudes towards PSRs and more contact with them than pharmacy students (*n* = 54) [32]. Similarly, in a large survey of mental health nursing students at two universities in the United Kingdom, 88% (305 out of 347) believed it was acceptable to receive some form of gift from industry [40]. Over half (57%) of the students believed that pharmaceutical representatives did not always give unbiased information, but thought that they and mental health nurses in general would be able to detect any bias. Only 20% believed that the clinical environment should be free of promotional objects. A study of 51 psychiatric nurses reported that an unspecified “majority” had received no guidance about working with the pharmaceutical industry [33].

Three industry-friendly articles [22,29,34], two of which were written by previous or current employees of the pharmaceutical industry [22,29], applaud the role of PSRs, noting their role in education. Whilst the authors' history of working for industry is described, this history is presented as a credential for, rather than risk to, the credibility of their claims. Willis, in an article on career options, recommends the pharmaceutical sales force as a career option for nurses and uncritically equates pharmaceutical sales to promoting solutions to patients [41].

### Concern about the pharmaceutical industry.

Thirteen of 31 articles express or report unequivocal concerns about the risks to practice presented by the pharmaceutical industry. Nursing professor Lisa Day's theoretical discussion of gift exchange argues that any gift, however small, places an obligation on recipients, resulting in unwanted debt. Such debt may be repaid by prescribers and those influencing them [42].

A similarly critical view of the industry is apparent in two general news articles reporting current events and describing concerns about drug companies' influences on nurses, voiced by a union leader in the first article, and by a range of nursing, pharmacy, and medical experts speaking to the UK House of Commons about drug company marketing strategies in the second [43,44]. Lecturers in mental health nursing Russell Ashmore and Neil Carver are particularly sceptical about supposed benefits of information provided by industry, and point out that mental health nurses may be particularly vulnerable to pharmaceutical company advertising and “clinical support” [18,40]. Nurse ethicist Nancy Crigger's assessment of pharmaceutical promotion and NP decision making offers a critique of the industry, and argues for guidelines to reduce potential harms, but is diluted by PSR Larry Bennison's counterargument in the same article [45].

Four nonsystematic narrative reviews address nursing education, and how guidelines and professional responsibilities may help to shift nursing education from commercial to professional sources of information [26,39,46,47]. Many of these articles point out the ethical challenges of pharmaceutical gift giving but offer few, if any, solutions. There is a comfortable sense that nurses, once alerted, will not be “caught” by marketing practices, that skills central to the nursing profession inherently provide nurses with the ability to evaluate information effectively [26,46], that ethics committees will give adequate guidance [48], and that guidelines or codes will prevent the problem [47,49]. A strongly worded debate between a sceptical NP and a PSR concludes with vague references to guidelines and raising awareness [45]. Sidiqi's letter to the editor of *The Nurse Practitioner* is just as strongly worded, but similarly, is quickly deflated by the editor's comments about NPs being unlikely to get “caught” in the same way as doctors [37,38].

Remarkably, none of the articles from the nursing literature reviewed here included author disclosure about possible competing interests. Over the past two decades, medical journals have increasingly required authors to declare competing interests, as these are recognised as potential sources of bias in the collection, analysis, and interpretation of data [50]. As we have seen, nurses are subject to many of the same conflicts of interest as doctors, yet the nursing literature lacks even this most basic means of detecting possible bias.

## Discussion

Nursing education fails to prepare graduates to deal with pharmaceutical promotion. From the scant empirical work available, many nurses would appear to accept promotional material uncritically. Nurses, just like doctors, might benefit from understanding marketing and persuasion [51].

Nurses should be encouraged to re-evaluate the educational benefits of promotional information, which is carefully selected, prone to bias, and hardly likely to be as beneficial as many believe [52,53]. Similarly, they should reconsider the presumed educational benefit and lack of bias in DTCA, as these presumptions have now been widely refuted [54–56]. Rather simplistically, many articles about nurses and DTCA have announced that nurses must be “cognizant” or “aware”—suggesting that by knowing the scope of the problem, and by working “with” the industry, nurses will be able to avoid complicity in unethical promotion [19,20,24,34,49].

This optimistic approach belies the fact that many nurses are not trained in critical appraisal, and appear to understand little of the mechanisms by which marketing strategies operate. Numerous studies have found that doctors, regardless of seniority, tend to have poor understanding of marketing, and of their own vulnerability, decision-making processes, and conflicts of interest [57–59]. Nurses are less well-studied in this regard, but are likely to have similar difficulties, exacerbated by their relatively meagre training in pharmacology, statistical inference, and critical appraisal.

### Understanding the nursing perspective.

Nursing's relationship to medicine may provide some insight into the lack of critical outcry in the nursing literature regarding pharmaceutical marketing. Direct-to-consumer advertising and the ubiquity of PSRs allow both patients and nurses to circumvent the doctor as source of authoritative knowledge. Information access, however biased, has thus become a source of independence for nurses and patients alike.

The importance of improving nurse–doctor relationships may underlie the prevalent belief among nurses that contact with the pharmaceutical industry is beneficial, opening channels of communication and providing information that enables them to interact with doctors on a more even footing. It seems likely that the ability to seek information from non-medical sources may be perceived by some nurses as a way to escape traditional medical dominance. The perception of DTCA as a means to redress this power imbalance is captured in the commentary of patient advocate Charles Inlander, who in 1991 opined in *Nursing Economic$* that DTCA is a tool by which patients could circumvent medical paternalism [21]. Disdain towards medical dominance is also apparent in NP publications, whose authors seem to be indignant that doctors get “courted” by the pharmaceutical industry, while NPs are ignored [36,38].

Both the doctor–patient and the doctor–nurse relationship have evolved in recent years. Ethicists Ezekiel Emanuel and Linda Emanuel have pointed out that the doctor–patient interaction has historically been paternalistic, where the doctor—with superior education and knowledge of human biology—was better positioned than patients to determine their best interest [60]. However, both nurses and patients now have greater access to information outside the clinical encounter, notably including DTCA, PSRs, and the Internet, accelerating changes to these relationships. However, transferring the power imbalance from a paternalistic to a commercial relationship is hardly the last word in liberation. This model purports to transfer autonomy, yet presents information designed to sell, rather than to enable rational treatment choices.

### The nursing profession's responsibility.

We propose a three-pronged approach including education, clinical policy, and research to aid nurses with the challenges of pharmaceutical promotion.

Firstly, nurses require training to understand and manage the impact of commercial activity, ideally well before post-graduate training [32]. Nurses already in the work force should receive continuing education on the range of interests promoted in health care, including those motivated by profit.

Little in their training provides nurses with adequate understanding of relevant fields such as pharmacology, epidemiology, public health, evidence-based medicine, critical appraisal, psychology, social science, management, and communications studies, to the extent that would allow them to reliably understand and manage commercial promotion [61]. Educational institutions and licensing authorities should ensure that their students or registrants receive training on the influences of commercialism and have unbiased resources to support prescribing and clinical decisions. Nurses will hopefully come to appreciate the vulnerability they share with doctors to the charms of the PSR.

Secondly, institutional guidelines, policy, and quality assurance should be developed to complement such education. The exclusion of commercial sponsorship from nursing education would leave a gap; careful consideration should be made about how this gap may be filled with information based on best practice and made transparent with respect to its inherent biases.

Policy makers and managers in health care must identify and prevent the intrusion of external interests in clinical decisions. The Stanford University initiative to ban PSRs from its hospitals is an interesting case in point. Whilst on the one hand, it makes bold steps towards curtailing the presence of the pharmaceutical industry in its facilities, on the other, it makes no specific reference to nurses [62]. Such policies should carefully consider the issues of sponsorship and gifts in reference to nurses as well as physicians.

The final prong in our strategy is to gain a better understanding of nurses' role in and influence on prescribing. In the United Kingdom, for example, a drive to nurse prescribing is part of the professionalisation of nursing, and is seen to enhance nursing's status, patient care, and the use of health care resources [63]. This prescribing practice places nurses in an analogous position vis-à-vis the pharmaceutical industry as their medical colleagues.

But it is not only prescribing nurses who must be the focus of these strategies. As we have described above, non-subscribing nurses play an under-appreciated role in prescription and administration choices. One of us (AJ), an experienced staff nurse, recalls guiding new house staff in the choice of treatment; the other (DBM) was grateful for such guidance as a medical intern.

Research is required into the mechanisms by which nurses influence medical prescribing, and the frequency of such influence in various settings. There are a myriad of likely ways in which their influence may be applied, from nurseled clinics where the decision to refer is in the nurse's hands, to drug cabinet stocking, treatment monitoring and assessment, and protocol development. Nurses must identify and acknowledge these roles, and the enormous responsibility they confer, to avoid being a soft target for commercial promotion.

## Conclusion

The pharmaceutical industry recognises nursing influence on medical prescribing and identifies nurses as a marketing target. The industry has had its eye on nurses and nurse practitioners for over a decade [64], and is heavily invested in wooing them [65]. Unfortunately, its success in this area has been at the expense of the health budget, evidence-based care, and nursing integrity. All three can and must be reclaimed.

## Abbreviations

DTCA: direct-to-consumer advertising
NP: nurse practitioner
PSR: pharmaceutical sales representative
